# Immunologic Gene Sets Reveal Features of the Tumor Immune Microenvironment and Predict Prognosis and Immunotherapy Response: A Pan-Cancer Analysis

**DOI:** 10.3389/fimmu.2022.858246

**Published:** 2022-04-14

**Authors:** Hongda Pan, Jingxin Pan, Pei Li, Jianghong Wu

**Affiliations:** ^1^ Department of Gastric Surgery, Fudan University Shanghai Cancer Center, Shanghai, China; ^2^ Department of Oncology, Shanghai Medical College, Fudan University, Shanghai, China; ^3^ Department of Hematology, The Second Affiliated Hospital of Fujian Medical University, Quanzhou, China; ^4^ Department of Breast Surgery, Fudan University Shanghai Cancer Center, Shanghai, China

**Keywords:** immunologic gene set, pan-cancer analysis, immunotherapy response, prognosis, CD8+ T-cell infiltration

## Abstract

In the treatment of cancer, anti-programmed cell death-1 (PD-1)/programmed cell death-ligand 1 (PD-L1) immunotherapy has achieved unprecedented clinical success. However, the significant response to these therapies is limited to a small number of patients. This study aimed to predict immunotherapy response and prognosis using immunologic gene sets (IGSs). The enrichment scores of 4,872 IGSs in 348 patients with metastatic urothelial cancer treated with anti-PD-L1 therapy were computed using gene set variation analysis (GSVA). An IGS-based classification (IGSC) was constructed using a nonnegative matrix factorization (NMF) approach. An IGS-based risk prediction model (RPM) was developed using the least absolute shrinkage and selection operator (LASSO) method. The IMvigor210 cohort was divided into three distinct subtypes, among which subtype 2 had the best prognosis and the highest immunotherapy response rate. Subtype 2 also had significantly higher PD-L1 expression, a higher proportion of the immune-inflamed phenotype, and a higher tumor mutational burden (TMB). An RPM was constructed using four gene sets, and it could effectively predict prognosis and immunotherapy response in patients receiving anti-PD-L1 immunotherapy. Pan-cancer analyses also demonstrated that the RPM was capable of accurate risk stratification across multiple cancer types, and RPM score was significantly associated with TMB, microsatellite instability (MSI), CD8+ T-cell infiltration, and the expression of cytokines interferon-γ (IFN-γ), transforming growth factor-β (TGF-β) and tumor necrosis factor-α (TNF-α), which are key predictors of immunotherapy response. The IGSC strengthens our understanding of the diverse biological processes in tumor immune microenvironment, and the RPM can be a promising biomarker for predicting the prognosis and response in cancer immunotherapy.

## Introduction

Anticancer immunotherapy, mainly immune checkpoint inhibitors (ICIs), has emerged as a new pillar in cancer management (1, 2). These treatments work by overcoming tumor-induced immunosuppression, thereby achieving immune-mediated tumor clearance (3). Although they are generally more effective and better tolerated than conventional and targeted therapies, many patients have innate or acquired resistance to immunotherapy (4).

The tumor immune microenvironment (TIME) has been shown to play a critical role in tumor development and influence clinical outcomes (5). Comprehensive analysis of the TIME can reveal the mechanisms of immunotherapy response and resistance, thus providing opportunities to improve survival outcomes and develop new therapeutic strategies (6). Gene expression profiling has become a mainstay in the research field of the TIME (7). However, due to its highly heterogeneous and dynamic nature, studies regarding the changes in an individual gene cannot precisely dissect the TIME. Generally, immune cell (IC) function is affected by a set of correlated genes rather than a single gene. Therefore, studies of gene sets may provide novel insights into cancer immunotherapy.

In this study, we assessed the enrichment changes in immunologic gene sets (IGSs) from the ImmuneSigDB in a cohort of patients with anti-PD-L1-treated metastatic urothelial cancer (IMvigor210). The correlation of IGS-based classification (IGSC) with clinical and immune characteristics was assessed. Finally, an IGS-based risk prediction model (RPM) for prognosis and the immunotherapy response was established, and its prognostic predictive ability was evaluated across multiple types of cancers.

## Materials and Methods

### Data Acquisition

IMvigor210 is a multicenter, single-arm, phase II trial to assess the safety and efficacy of atezolizumab (a PD-L1 inhibitor) in patients with locally advanced and metastatic urothelial carcinoma (8, 9). After procuring the Creative Commons 3.0 License, we obtained the transcriptome RNA sequencing (RNA-seq) and detailed clinical annotations from IMvigor210CoreBiologies (http://research-pub.gene.com/IMvigor210CoreBiologies). Transcriptome and clinical data of Liu’s cohort were extracted from the Supplementary File of their articles (10). Clinicopathological information and microarray expression profiling data of immunotherapy cohorts (GSE78220 and GSE91061).

Transcriptome RNA-seq and somatic mutation data and the corresponding clinicopathological information of 5,265 patients with the 12 most common cancers [bladder carcinoma (BLCA), breast invasive carcinoma (BRCA), cervical squamous cell carcinoma and endocervical adenocarcinoma (CESC), colon adenocarcinoma (COAD), esophageal carcinoma (ESCA), liver hepatocellular carcinoma (LIHC), lung adenocarcinoma (LUAD), lung squamous cell carcinoma (LUSC), prostate adenocarcinoma (PRAD), rectum adenocarcinoma (READ), stomach adenocarcinoma (STAD), and thyroid carcinoma (THCA)] were downloaded from The Cancer Genome Atlas (TCGA) database (https://portal.gdc.cancer.gov). Pan-cancer microsatellite instability (MSI) scores were obtained from the article by Bonneville et al. (11).

### Immunologic Gene Set and Gene Set Variation Analysis

ImmuneSigDB is a manually annotated compendium of ∼5,000 gene sets from diverse cell states, experimental manipulations, and genetic perturbations in immunology (12). IGS of ImmuneSigDB (c7. ImmuneSigDB. v7.4) was downloaded from Molecular Signatures Database (MSigDB). The enrichment score (ES) for each IGS in each sample was analyzed by GSVA with the “GSVA” package in R. GSVA is a popular enrichment algorithm, which was extensively utilized in medical studies (13–17).

### Immunologic Gene Set-Based Classification

According to the ES of IGS, subtypes of IMvigor210 cohort were identified using nonnegative matrix factorization (NMF) with “NMF” package in R (18). The optimal number of clusters (K) was generated using “factoextra” package.

### Immune Cell Infiltration Analysis

CIBERSORT (https://cibersortx.stanford.edu/) is a computational method used to quantify cell fractions from bulk tissue gene expression profiles (19). We used CIBERSORT to estimate the proportion of 22 types of ICs in IMvigor210 and pan-cancer cohorts.

### Immunotherapy Response Prediction

Tumor immune dysfunction and exclusion (TIDE) (http://tide.dfci.harvard.edu/) is a computational framework developed to evaluate the potential of tumor immune escape from the gene expression profiles of cancer samples (20). The TIDE score computed for each tumor sample can serve as a surrogate biomarker to predict the response to immune checkpoint blockade for multiple types of cancers.

### Statistical Analysis

Statistical analyses were conducted using the R software (version 4.1.0) and the Sangerbox tools (http://www.sangerbox.com/tool). Continuous variables were presented as standard error of the mean and were compared using Student’s t-test or Wilcoxon rank sum test. Categorical data were compared using the chi-square test. Univariate and multivariate Cox proportional hazards regression analysis using the “survival” package. A least absolute shrinkage and selection operator (LASSO) regression model was performed with “glmnet” and “survival” packages. Kaplan–Meier survival analysis with log-rank test was performed with the R package “survminer”. Differential expression analysis was performed with the “limma” package. Statistical significance was set at *P* < 0.05 and shown as *P < 0.05, **P < 0.01, and ***P < 0.001.

## Results

### Construction of an Immunologic Gene Set-Based Classification

**Figure 1:**
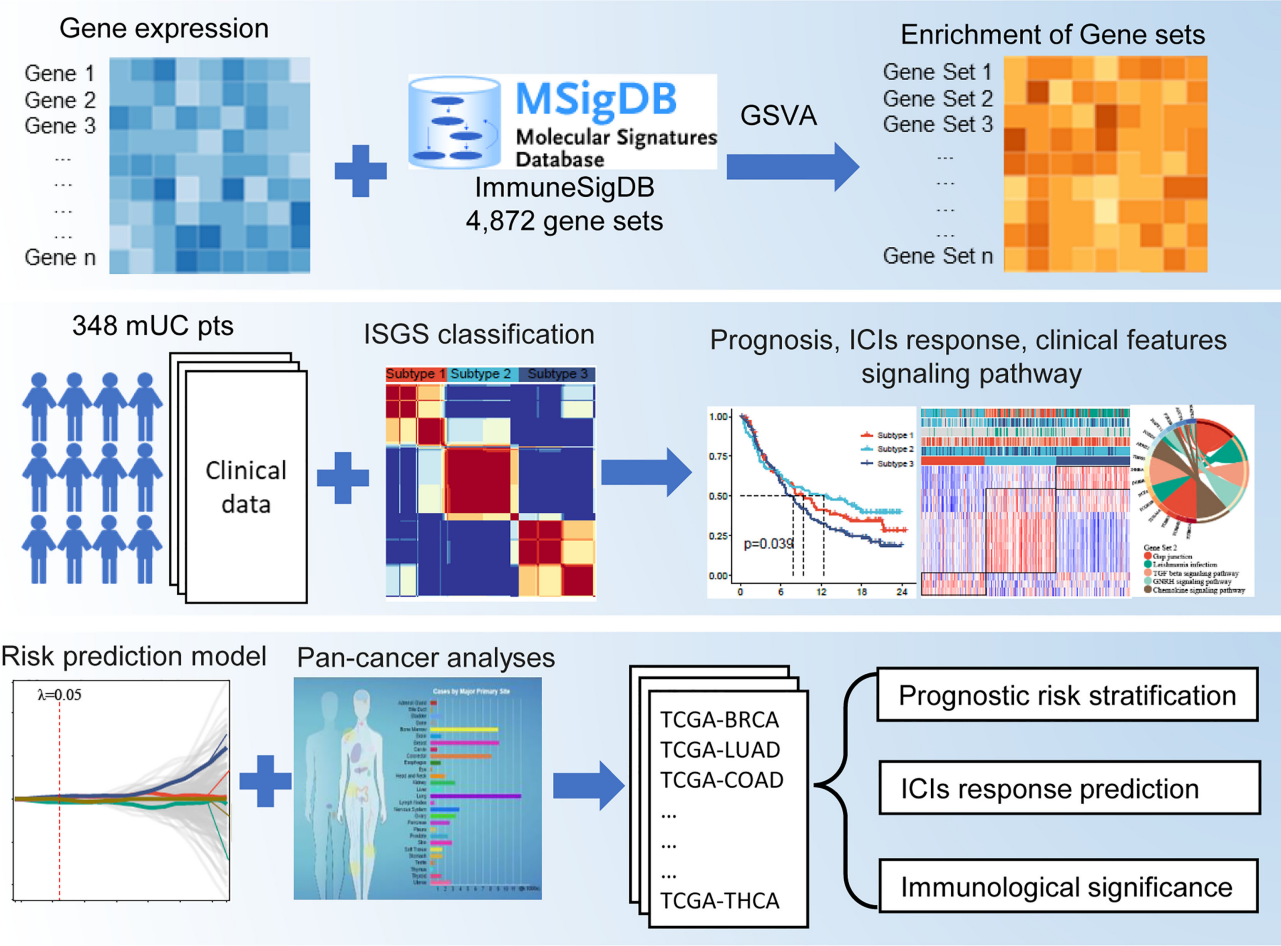
Graphic abstract of this study.

**Figure 2:**
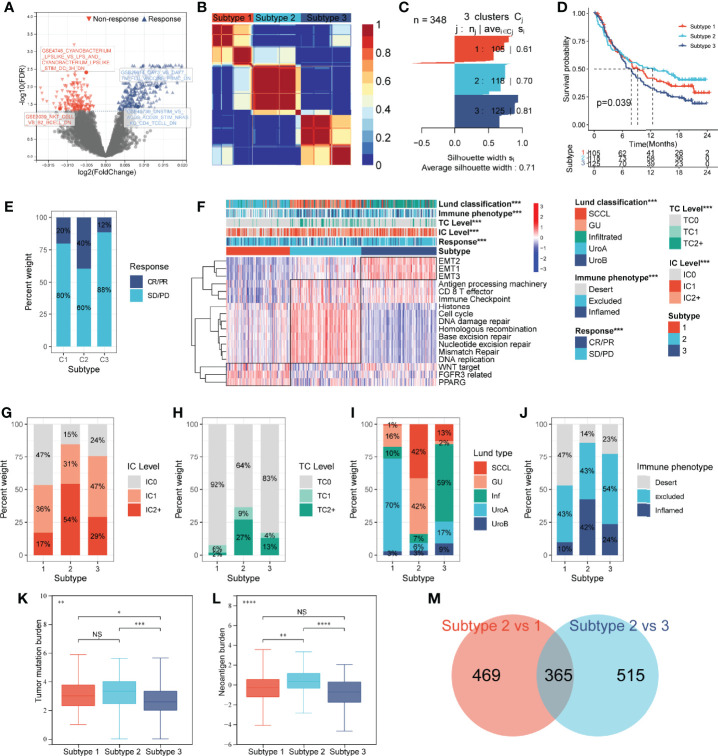
Construction of an IGS-based classification (IGSC). **(A)** A volcano plot showed the differentially expressed gene sets between response and non-response groups in IMvigor210 cohort. **(B)** IMvigor210 cohort was classified into three subtypes. **(C)** The silhouette width value for the classification was 0.71, suggesting a fine match between a sample and its identified subtype. **(D)** Kaplan–Meier survival analysis showed that Subtype 2 had the best prognosis, Subtype 3 had the worst prognosis, and Subtype 1 had an intermediate prognosis. **(E)** The response rate to PD-L1 immunotherapy was significantly higher in Subtype 2 than those in Subtypes 3 and 1. **(F)** A heatmap showed the correlation between IGSC subtypes and clinical and immune features. **(G–J)** The proportions of IC levels (IC0, IC1, and IC2+), TC levels (TC0, TC1, and TC2+), Lund classifications (SCCL, GU, inf, UroA, and UroB), and immune phenotypes (inflamed, excluded, and desert) in different IGSC subtypes. **(K, L)** Tumor mutational burden and neoantigen burden in different IGSC subtypes. **(M)** A Venn plot shows the intersected differential gene sets between Subtypes 2 and 1 and between Subtypes 2 and 3. *P < 0.05, **P < 0.01, ***P < 0.001, ****P < 0.0001; NS, nonsignificant; IGS, immunologic gene sets; PD-L1, programmed cell death-ligand 1; IC, immune cells; TC, tumor cells; Inf, infiltrated; SCCL, squamous cell carcinoma-like; UroA, urothelial-like A; UroB, urothelial-like B.

The flowchart of this study is shown in **Figure 1**. A total of 4,872 IGSs were obtained from ImmuneSigDB. The ESs for the IGSs from 348 urothelial carcinoma specimens in the IMvigor210 cohort were computed using GSVA based on transcriptome RNA-seq data. Patients who responded to treatment with a complete response (CR; N = 25) or partial response (PR; N = 43) were categorized as responders, and patients who displayed stable (SD; N = 63) or progressive disease (PD; N = 167) were categorized as non-responders. Differential expression analysis revealed that ESs of 1,349 gene sets were significantly different between responders and non-responders (false discovery rate <0.05); 744 gene sets were upregulated in the responder group, and 605 gene sets were significantly upregulated in the non-responder group (**Figure 2A**). The univariate Cox regression model showed that 367 gene sets were significantly associated with prognosis.

Based on the prognostic IGS, the IMvigor210 cohort was classified into three distinct subtypes according to the optimal number of subtypes (K) as defined by the “factoextra” package (**Figure 2B**), namely, Subtype 1 (N = 105), Subtype 2 (N = 118), and Subtype 3 (N = 125). The silhouette width value for the classification was 0.71 (**Figure 2C**), suggesting a fine match between a sample and its identified subtype. Kaplan–Meier survival analysis showed that Subtype 2 had the best prognosis, Subtype 3 had the worst prognosis, and Subtype 1 had an intermediate prognosis (**Figure 2D**). Additionally, the response rate to PD-L1 immunotherapy was significantly higher in Subtype 2 (40%) than those in Subtypes 3 (12%) and 1 (20%) (**Figure 2E**). The above results suggest that IGSC can be effective for distinguishing patients with different prognoses and can predict the efficacy of anti-PD-L1 immunotherapy.

### Immunologic Gene Set-Based Classification Subtypes Correlated With Clinical and Immune Features

As shown in the heatmap in **Figure 2F**, IGSC was significantly correlated with PD-L1 expression, Lund classification, and immune phenotype. Specifically, Subtype 2 had higher PD-L1 expression levels in both ICs and tumor cells (TCs). For ICs, the proportion of IC2+ in Subtype 2 (54%) was significantly higher than those in Subtypes 1 (17%) and 3 (29%) (**Figure 2G**). The same was also observed for TCs, with a significantly higher percentage of TC2+ (27%) in Subtype 2 than that in Subtypes 1 (2%) and 3 (13%) (**Figure 2H**). In addition, we found that about 70% of Subtype 1 was UroA type, and about 60% of Subtype 3 was Inf type; the proportions of GU type (42%) and SCCL type (42%) in Subtype 2 were significantly higher than those in the other two subtypes (**Figure 2I**). Next, we compared the IGSC subtypes to immune phenotypes and found that Subtype 2 had the highest proportion of immune-inflamed phenotype (42%) and the lowest proportion of the immune-desert phenotype (14%) (**Figure 2J**). Furthermore, Subtype 2 had higher levels of tumor mutational burden (TMB) (**Figure 2K**) and tumor neoantigen burden (TNB) (**Figure 2L**) than Subtypes 1 and 3. Several prognostic favorable signatures, including CD8+ T effector, immune checkpoint, antigen processing machinery (APM), DNA damage repair (DDR), and mismatch repair (MMR), cell cycle, and DNA replication, were significantly upregulated in Subtype 2. In contrast, oncogenic signatures and epithelial–mesenchymal transition (EMT) signaling pathways, were significantly upregulated in Subtype 3, which explains why Subtype 3 had the worst prognosis. Subtype 1 was related to the activation of signaling pathways, such as Peroxisome Proliferator Activated Receptor Gamma (PPARG), Fibroblast Growth Factor Receptor 3 (FGFR3) related, and Wingless/Integrated (WNT) target (**Figure 2A**).

### Immunologic Gene Set-Based Classification Differential Gene Sets and the Construction of a Risk Prediction Model

**Figure 3:**
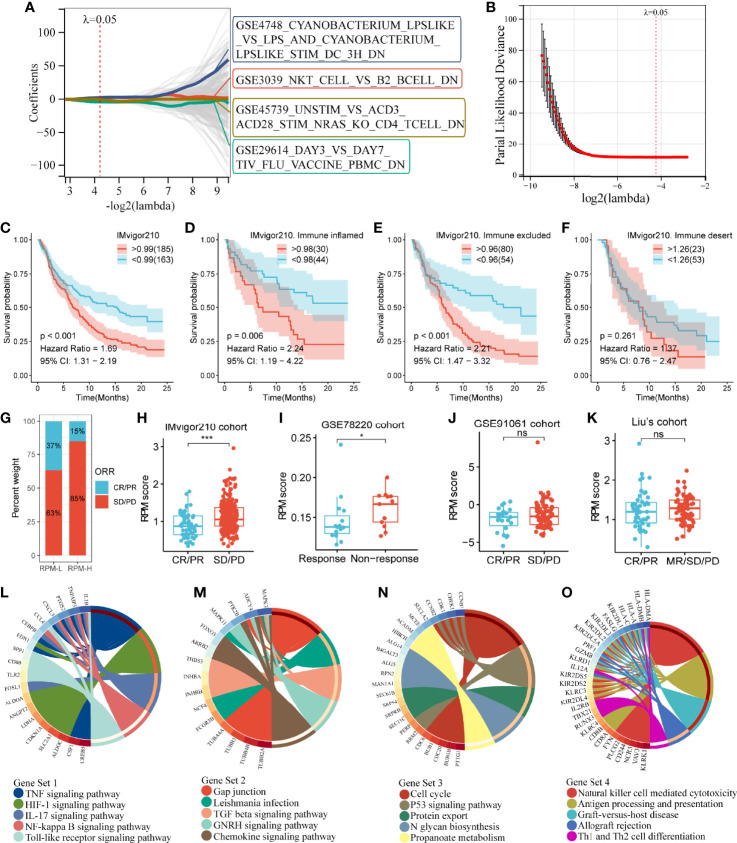
Construction of an IGS-based risk prediction model (RPM). **(A, B)** LASSO regression analysis identified four gene sets to construct an RPM. **(C)** Kaplan–Meier survival analysis showed that RPM-L had a better prognosis than RPM-H. **(D–F)** Subgroup analysis based on immune phenotypes revealed the difference in survival between RPM-H and RPM-L groups in immune-inflamed (**D**), -excluded **(E)** and -desert **(F)** subtypes. **(G)** The response rate to PD-L1 immunotherapy was significantly higher in the RPM-L than that in the RPM-H. **(H)** Patients with CR/PR had a lower RPM score than those with SD/PD. **(I–K)** The predictive ability of RPM for immunotherapy response was validated in GSE78220, GSE91061, and Liu’s cohort. **(L–O)** KEGG pathway analysis of the genes in Gene Sets 1–4. *P < 0.05, ***P < 0.001; ns, nonsignificant; RPM, risk prediction model; LASSO, least absolute shrinkage and selection operator; PD-L1, programmed cell death-ligand 1; CR, complete response; PR, partial response; SD, stable disease; PD, progressive disease; KEGG, Kyoto Encyclopedia of Genes and Genomes.

The above results indicated that Subtype 2 had significantly better prognosis and immunotherapy response than Subtypes 1 and 3. To explore the mechanisms underlying these differences, we performed a differential analysis of gene sets among the IGSC subtypes. There were 834 differential gene sets between Subtypes 1 and 2 and 880 differential gene sets between Subtypes 3 and 2. Taking the intersection, we obtained 365 differential gene sets (**Figure 2M**). These gene sets were applied to a LASSO regression analysis, and finally an RPM consisting of four gene sets was constructed (**Figures 3A, B** **)**. The four gene sets are hereafter referred to as Gene Set 1 (GSE3039_NKT_CELL_VS_B2_BCELL_DN), Gene Set 2 (GSE4748_CYANOBACTERIUM _LPSLIKE_VS _LPS_AND_CYANOBACTERIUM_ LPSLIKE_STIM_DC_3H_DN), Gene Set 3 (GSE29614_DAY3_VS_ DAY7_TIV_FLU_VACCINE _PBMC_DN), and Gene Set 4 (GSE45739_UNSTIM_VS_ACD3_ACD28_ STIM_NRAS_KO_CD4_ TCELL_DN). Genes included in Gene Sets 1–4 were summarized in **Table S1**. High expression of Gene Sets 1 and 2 was associated with worse prognosis, while high expression of Gene Sets 3 and 4 was associated with better survival (**Figures S1A–D**).

By calculating the sum of the products of the ES and coefficients for each gene set, we could quantify the prognosis of each patient. RPM score = (Gene Set 1 × 7.47) + (Gene Set 2 × 5.73) + (Gene Set 3 × -6.00) + (Gene Set 4 × -6.28). We divided the IMvigor210 cohort into low-risk (RPM-L, N = 163) and high-risk (RPM-H, N = 185) groups based on the best cutoff values calculated using the “Survminer” package in R. Clinicopathological features of the RPM-L and RPM-H groups in IMvigor210 cohort are summarized in **Table S2**. Kaplan–Meier survival analysis showed that the prognosis of the RPM-H group was significantly worse than that of the RPM-L group [**Figure 3C**; *P* < 0.001, hazard ratio (HR) = 1.69]. We performed subgroup analysis based on immune phenotypes, and the IMvigor210 cohort was divided into three subgroups, namely, immune inflamed (n = 74), immune excluded (n = 134), and immune desert (n = 76). The RPM-H group showed worse prognosis compared to the RPM-L group in immune inflamed (HR = 2.24, p = 0.006; **Figure 3D**) and immune excluded (HR = 2.21, p < 0.001; **Figure 3E**) subgroups. Although a similar trend was also seen in the immune desert subgroup, the difference in survival did not reach statistical significance (HR = 1.37, p = 0.261; **Figure 3F**). The proportion of CR/PR in the RPM-L group (37%) was significantly higher than that in the RPM-H group (15%) (**Figure 3G**), and the RPM score in the SD/PD group was significantly higher than that in the CR/PR group (**Figure 3H**). To validate the predictive ability of RPM for immunotherapy response, we calculated RPM scores in GSE78220 (28 melanoma patients treated with anti-PD-1 inhibitor), GSE91061 (105 melanoma patients treated with anti-PD-1 inhibitor), and Liu’s cohort (121 melanoma patients treated with anti-PD-1 inhibitor). We found that non-responders to immunotherapy had significantly higher RPM than responders in the GSE78220 cohort (*P* = 0.041; **Figure 3I**). A minor difference in RPM score between responder and non-responder can also be seen in GSE91061 (**Figure 3J**) and Liu’s cohort (**Figure 3K**), but the difference did not reach statistical significance.

Kyoto Encyclopedia of Genes and Genomes (KEGG) pathway analyses showed that Gene Set 1 was associated with TNF, HIF-1, IL-17, NF-kappa B, and Toll-like receptor signaling pathways (**Figure 3L**); genes in Gene Set 2 were significantly enriched in TGF-beta and chemokine signaling pathways (**Figure 3M**). Gene Set 3 was correlated with cell cycle and the p53 signaling pathway (**Figure 3N**); and Gene Set 4 was related to natural killer cells, Th1 and Th2 cells, and antigen processing and presentation (**Figure 3O**).

### Correlation of Risk Prediction Model With Clinical Characteristics

**Figure 4:**
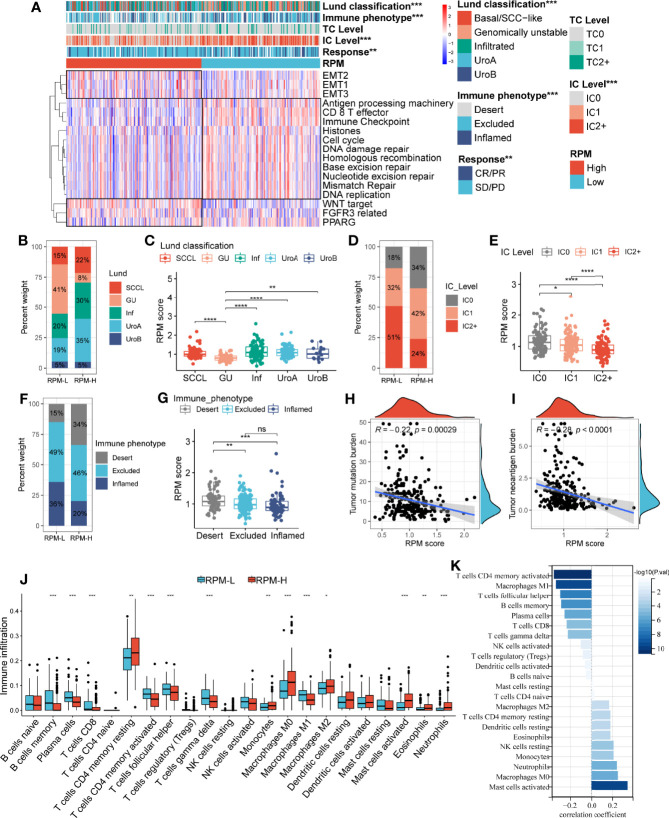
Correlation of the RPM with clinical characteristics and immune cell infiltration. Panel **A** showed the correlation between IGSC subtypes and clinical and immune features. **(B, D, F)** The proportions of Lund classifications, IC levels, and immune phenotypes in different RPM groups. **(C, E, G)** The RPM scores in different Lund classifications, IC levels, and immune phenotypes. **(H, I)** Correlations of RPM scores with TMB and TNB. **(J)** Immune cell infiltration in RPM-L and RPM-H groups. **(K)** Correlation between RPM scores and density of infiltrating immune cell. *P < 0.05, **P < 0.01, ***P < 0.001, ****P < 0.0001; NS, nonsignificant; RPM, risk prediction model; IGSC, immunologic gene sets-based classification; IC, immune cells; TMB, tumor mutation burden; TNB, tumor neoantigen burden.

Similar to the IGSC, the RPM was significantly correlated with Lund classification, immune phenotype, and IC level (**Figure 4A**) but not with TC level (**Figures S1E, F**). In the RPM-L group, more than 40% were GU type, while in the RPM-H group, only 8% were GU type (**Figure 4B**), and of the five types, the GU type had the lowest RPM score (**Figure 4C**). The proportion of IC2+ in the RPM-L group was about twice that in the RPM-H group, while the proportion of IC0 was only half of that in the RPM-H group (**Figure 4D**). The RPM score of the IC2+ was significantly lower than that of the IC0 and IC1 (**Figure 4E**). The proportion of the immune-desert phenotype was significantly higher in the RPM-H group (35% vs. 14%; **Figure 4F**), while the proportion of the immune-inflamed phenotype was significantly lower in the RPM-H group (21% vs. 38%) than that in the RPM-L group. The immune-desert phenotype had the highest RPM score, while the immune-inflamed phenotype had the lowest RPM score (**Figure 4G**). In addition, RPM scores were also significantly negatively correlated with both TMB and TNB (**Figures 4H, I**).

Correlations between RPM scores and core signaling pathways were also analyzed, and RPM-L was significantly correlated with prognosis-favorable biological pathways, including CD8+ T effector, immune checkpoint, APM, and DDR, while RPM-H was associated with prognosis-unfavorable signatures, such as EMT, WNT, FGFR3, and PPARG (**Figure 4A**).

### Correlation of Risk Prediction Model With Immune Cell Infiltration and Expression of Immune Checkpoints

**Figure 5:**
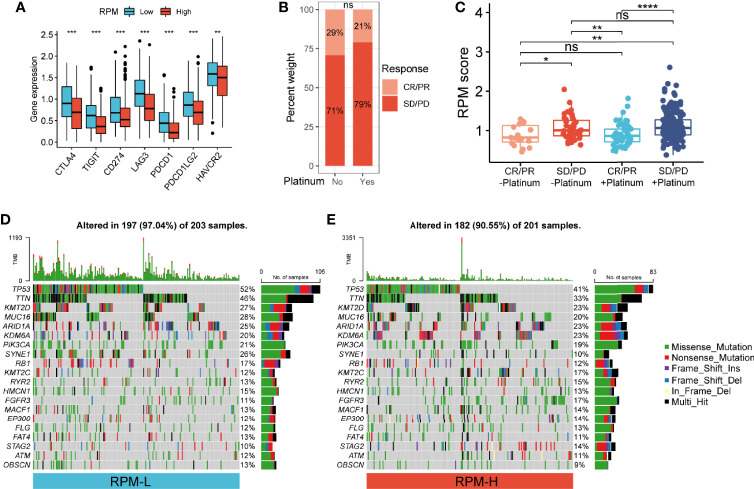
Correlation of RPM with immune checkpoints, platinum-based chemotherapy, and somatic mutation. **(A)** RPM-L group had significantly higher expression levels of immune checkpoint molecules compared to those in the RPM-H group. **(B)** Immunotherapy response in patients who received platinum-based chemotherapy and those who did not. **(C)** RPM score in CR/PR and SD/PD groups with or without platinum-based chemotherapy. **(D, E)** Somatic mutation landscape of the 20 most frequently mutated genes in RPM-L **(D)** and RPM-H **(E)** groups in TCGA-BLCA cohort *P < 0.05, **P < 0.01, ***P < 0.001, ****P < 0.0001; NS, nonsignificant. RPM, risk prediction model; CR, complete response; PR, partial response; SD, stable disease; PD, progressive disease; TCGA, The Cancer Genome Atlas; BLCA, bladder cancer.

The infiltration of 22 types of ICs in the IMvigor210 cohort was analyzed using the CIBERSORT package. We found denser infiltrations of memory B cells, plasma cells, CD8 T cells, activated memory CD4 T cells, follicular helper T cells, gamma delta T cells, and M1 macrophages in the RPM-L group. In contrast, infiltration of resting memory CD4 T cells, monocytes, M0 macrophages, M2 macrophages, and activated mast cells, eosinophils, and neutrophils was denser in the RPM-H group (**Figure 4J**). Correlation analysis showed that RPM score was negatively correlated with CD8+ T cells, CD4+ T cells, and B cells while positively correlated with macrophages, mast cells, and neutrophils (**Figure 4K**). In addition, we compared the expression of immune checkpoint molecules, including CD274, PDCD1, PDCD1LG2, CTLA4, HAVCR2, LAG3, and TIGIT in the RPM-L and RPM-H groups. We found that the RPM-L group had significantly higher expression levels of immune checkpoint molecules compared to those in the RPM-H group (**Figure 5A**).

### Correlation of Risk Prediction Model With Platinum-Based Chemotherapy

Of the 348 patients with metastatic urothelial carcinoma (mUC), 272 received platinum-based chemotherapy. We compared the administration of platinum with immunotherapy response and found no significant difference in immunotherapy response in patients who received platinum-based chemotherapy (21%) vs. those who did not (29%) (*P* = 0.22; **Figure 5B**). Non-responders to immunotherapy had higher RPM score than responders, regardless of prior platinum-based chemotherapy. However, there was no significant difference in RPM score between patients who received platinum-based chemotherapy and those who did not (**Figure 5C**).

### Correlation of Risk Prediction Model With Somatic Mutation in Bladder Carcinoma

We analyzed the simple nucleotide variation data of TCGA-BLCA dataset to characterize somatic mutations in the RPM-H and RPM-L groups. We found that the overall mutation rate was significantly higher in the RPM-L group (97.04% vs. 90.55%). Compared with the RPM-H group, the mutation rate of the 20 most frequently mutated genes (such as *TP53*, *TTN*, *ARID1A*, *PIK3CA*, *FGFR3*, etc.) was higher in the RPM-L group (**Figures 5D, E**).

### Risk Prediction Model Can Predict Prognosis Across Pan-Cancer

**Figure 6:**
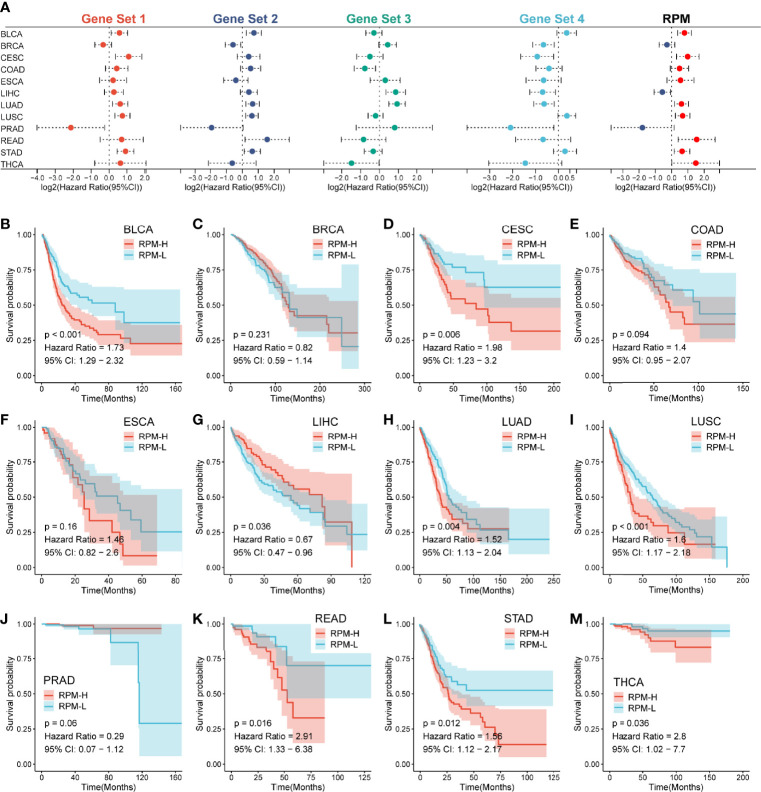
RPM predicts prognosis across multiple cancer types. **(A)** A forest plot showed the HR of Gene Sets 1–4 and RPM across 12 types of cancers. **(B–M)** Kaplan–Meier analysis showed differences in survival between RPM-L and RPM-H across 12 types of cancers; RPM, risk prediction model; HR, hazard ratio.

To further assess the general applicability of the RPM in predicting prognosis, we validated its prognostic predictive ability for the 12 most common cancers. We found that Gene Sets 1 and 2 had HRs >1 for most of the 12 tumors, suggesting that they are unfavorable prognostic indicators. In contrast, Gene Sets 3 and 4 had HRs <1 for most of the 12 tumors, indicating that they are favorable prognosis factors (**Figure 6A**). The prognosis of the RPM-H group was worse than that of the RPM-L group for 9 out of 12 types of cancers (BLCA, CESE, COAD, ESCA, LUAD, LUSC, READ, STAD, and THCA) (**Figures 6B, D–F, H, I, K–M**), and this difference in survival was not significant in BLCA, LIHC and PRAD (**Figures 6C, G, J**).

### Risk Prediction Model Predicts Immunotherapy Response Across Pan-Cancer

**Figure 7:**
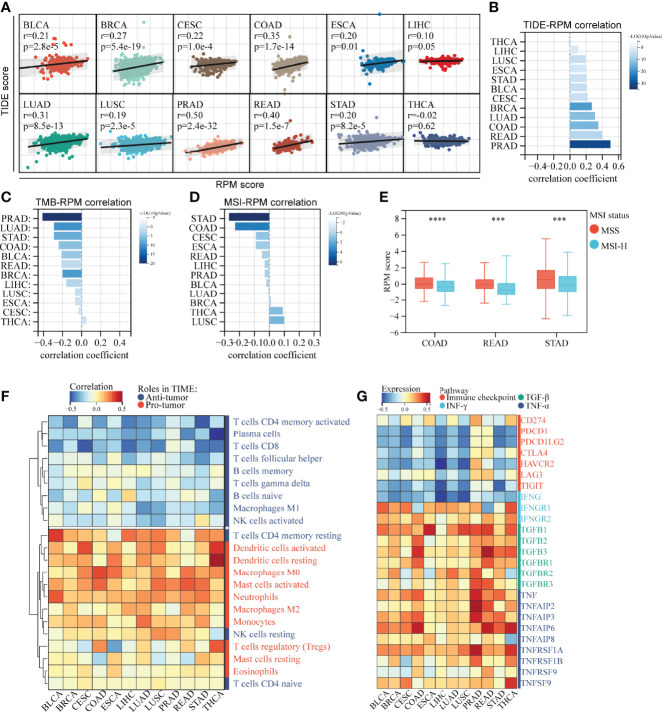
RPM predicts immunotherapy response across multiple cancer types. **(A, B)** RPM scores were generally positively associated with TIDE scores across multiple cancer types. **(C)** RPM scores were generally negatively associated with TMB across multiple cancer types. **(D)** Correlation between RPM scores and MSI across multiple cancer types. **(E)** RPM scores were higher in patients with MSS than in those with MSI-H in COAD, READ, and STAD. **(F)** Correlation of RPM with density of infiltrating immune cells across 12 types of cancers. **(G)** Correlation of RPM with immune checkpoints, IFN-γ, TGF-β, and TNF-α families across 12 types of cancers. ***P < 0.001, ****P < 0.0001; RPM, risk prediction model; TIDE, tumor immune dysfunction and exclusion; TMB, tumor mutation burden; MSI, microsatellite instability; MSS, microsatellite stability; COAD, colon cancer; READ rectal cancer; STAD, stomach cancer; IFN-γ, interferon-γ; TGF-β, transforming growth factor-β; TNF-α, tumor necrosis factor-α.

Based on their expression profiles, a TIDE score was calculated for 5,265 patients in pan-cancer cohorts. The RPM score was positively correlated with TIDE in 11 out of 12 cancers, except for THCA, which has a nonsignificant negative correlation (**Figures 7A, B**), implying that as the RPM score increased, the greater the likelihood of immune escape, and the less likely a patient would benefit from immunotherapy. In addition, RPM scores were negatively associated with TMB in 12 types of cancers (**Figure 7C**, **Figure S1G**). Moreover, RPM score was negatively correlated with MSI score in STAD, COAD, CESE, ESCA, and READ (**Figure 7D**). It is well known that MSI plays important roles in the carcinogenesis of gastrointestinal cancers, such as COAD, READ, and STAD. We found that patients with microsatellite stability (MSS) in COAD, READ, and STAD had significantly higher RPM scores than those with MSI-H (**Figure 7E**).

Correlation analysis of infiltrating ICs and RPM scores suggested that in most of the 12 tested cancers, RPM scores were significantly negatively correlated with the infiltration of T cells (except for resting CD4+ memory T cells and regulatory T cells) and B cells and were positively correlated with dendritic cells, macrophages, mast cells, and monocytes (**Figure 7F**). Moreover, RPM scores were negatively correlated with IFNG and immune checkpoint molecules, such as PD-1 (PDCD-1), PD-L1 (CD274), PD-L2 (PDCD1LG2), and CTLA4. In contrast, RPM scores were positively correlated with IFNGR1, TGF-β, and TNF-α families, which play pro-tumor roles in TIME (**Figure 7G**).

## Discussion

In this study, we identified three distinct subtypes and developed an RPM in the IMvigor210 cohort through a series of comprehensive analyses of ~5,000 IGSs from ImmuneSigDB. Previous studies have explored biomarkers for predicting immunotherapy response, including PD-L1 expression (21), TMB (22), MSI (23), and viral infection (24). In recent years, much effort has been devoted to developing genomic biomarkers of prognosis and response for patients receiving immunotherapy (25, 26). However, most of these studies are based on analyses of individual genes. In our study, we focused on sets of immunologic genes rather than individual genes, which will improve our understanding of the overall function of ICs. Based on prognostic IGSs, the IMvigor210 cohort was divided into three distinct subtypes. Notably, Subtype 2 had the best prognosis and the highest immunotherapy response rate. A previous study reported that PD-L1 expression on ICs was significantly associated with immunotherapy response (27). We found that the proportion of IC2+ was significantly higher in Subtype 2 than those in Subtypes 1 and 3. According to the antitumor immune response status, Chen and Mellman (28) proposed that cancers can be classified into three phenotypes: immune-desert, immune-excluded, and immune-inflamed types. Of these, the immune-inflamed type is the most sensitive to immunotherapy. Our study revealed that Subtype 2 group had the highest proportion of the inflamed phenotype and the lowest proportion of the desert phenotype, indicating high sensitivity to ICI therapy. CD8+ T cells are important in tumor immunity, as they can clear tumors *via* multiple mechanisms (29). Numerous studies have shown that high infiltration of CD8+ T cells in the TIME is associated with good prognosis in many malignancies (30, 31). In our study, we found that the CD8+ T effector signature was significantly upregulated in Subtype 2, which explains why Subtype 2 had a higher response and better prognosis. These results open new avenues for exploring the immune response and escape mechanisms of cancer immunotherapy.

There were 834 and 880 differential gene sets between Subtypes 1 and 2 and between Subtypes 3 and 2, respectively. Although there are also many differentially expressed IGSs (n = 1,771) between Subtypes 1 and 3, we found that both subtypes have poor prognosis and poor response to immunotherapy. The two subtypes did not show significant differences in clinical features such as immunotherapy response and TNB. Compared with Subtypes 1 and 3, Subtype 2 has the best prognosis and the highest immunotherapy response rate. It has distinct clinical, molecular, and immune correlates from Subtypes 1 and 3. Therefore, we only compared the differentially expressed IGSs between Subtype 1 and Subtype 2 and between Subtype 3 and Subtype 2 to elucidate the underlying mechanism by which subtype 2 is superior to Subtypes 1 and 3.

Four gene sets were identified that were used to construct an IGS-based RPM: Gene Set 1 contains genes that are downregulated in NKT cells compared to B2 B lymphocytes, Gene Set 2 contains genes that are downregulated in monocyte-derived dendritic cells, Gene Set 3 contains genes that are downregulated in a comparison of peripheral blood mononuclear cells collected from a TIV influenza vaccinee at day 3 post-vaccination vs. those collected at day 7 post-vaccination, and Gene Set 4 contains genes that are downregulated in CD4 T cells with NRAS knockout. These gene sets provide novel insights into the functional diversity of the TIME and thus provide potential biomarkers and therapeutic targets for cancer management. The four-gene set RPM effectively predicted prognosis and immunotherapy response in patients receiving anti-PD-L1 immunotherapy in the IMvigor210 cohort. Moreover, pan-cancer analyses showed that the RPM was capable of accurate risk stratification across multiple cancer types, indicating its broad applicability. Among the 12 most common cancers, RPM exhibited opposite prognostic outcomes in LIHC and PRAD compared to the other 10 cancers, and this may be due to their organ-specific immune environment (32). The difference in survival between the RPM-H and RPM-L groups for BRCA was not significant possibly due to its high overall survival and low mortality rate.

IMvigor210 provides high-quality gene expression data and complete clinical data (8); it is therefore ideal for construction and validation of molecular classification and RPM. Additionally, the predictive ability of RPM was validated in multiple types of cancers. Our study demonstrated that the RPM scores across 12 types of cancers were significantly associated with TMB, MSI, CD8+ T-cell infiltration, immune checkpoint molecules (PD-1/PD-L1, CTLA4), and cytokine (IFN-γ, TGF-β, and TNF-α) expression, which are key predictors of immunotherapy response. Therefore, we believe that this RPM has great potential to predict the response to immunotherapy in various types of cancers.

## Conclusion

In summary, we constructed the IGSC that may provide novel insights into the relationship between immunologic processes and features of TIME. Moreover, we developed a robust RPM that can accurately predict the prognosis and response to immunotherapy in patients with mUC, and its predictive ability was validated across multiple cancer types. IGSC and RPM can serve as useful tools for developing a novel strategy for cancer immunotherapy.

## Data Availability Statement

The original contributions presented in the study are included in the article/**Supplementary Material**. Further inquiries can be directed to the corresponding author.

## Author Contributions

Conception and design: HP and JW. Acquisition of data: PL. Writing, review, and revision of the article: HP, JP, and PL. Analysis and interpretation of data: HP and JP. Development of methodology: HP and JP. All authors contributed to the article and approved the submitted version.

## Funding

This study was funded by the National Natural Science Foundation of China (81902424).

## Conflict of Interest

The authors declare that the research was conducted in the absence of any commercial or financial relationships that could be construed as a potential conflict of interest.

## Publisher’s Note

All claims expressed in this article are solely those of the authors and do not necessarily represent those of their affiliated organizations, or those of the publisher, the editors and the reviewers. Any product that may be evaluated in this article, or claim that may be made by its manufacturer, is not guaranteed or endorsed by the publisher.

## Supplementary Material

Supplementary Figure 1 Prognostic and immunologic relevance of RPM. **(A–D)** Kaplan–Meier analyses of Gene Sets 1–4 used to construct the RPM. **(E)** The TC levels were not significantly different between RPM-L and RPM-H groups. **(F)** RPM scores were not significantly different among different TC levels. **(G)** Correlation of RPM scores with TMB across 12 cancer types. ns, nonsignificant.
