# Supplementary figures and images for: Immunologic Gene Sets Reveal Features of the Tumor Immune Microenvironment and Predict Prognosis and Immunotherapy Response: A Pan-Cancer Analysis
Source: Front Immunol. 2022 Apr 14;13:858246. doi: 10.3389/fimmu.2022.858246 (PMC9046667; doi:10.3389/fimmu.2022.858246)

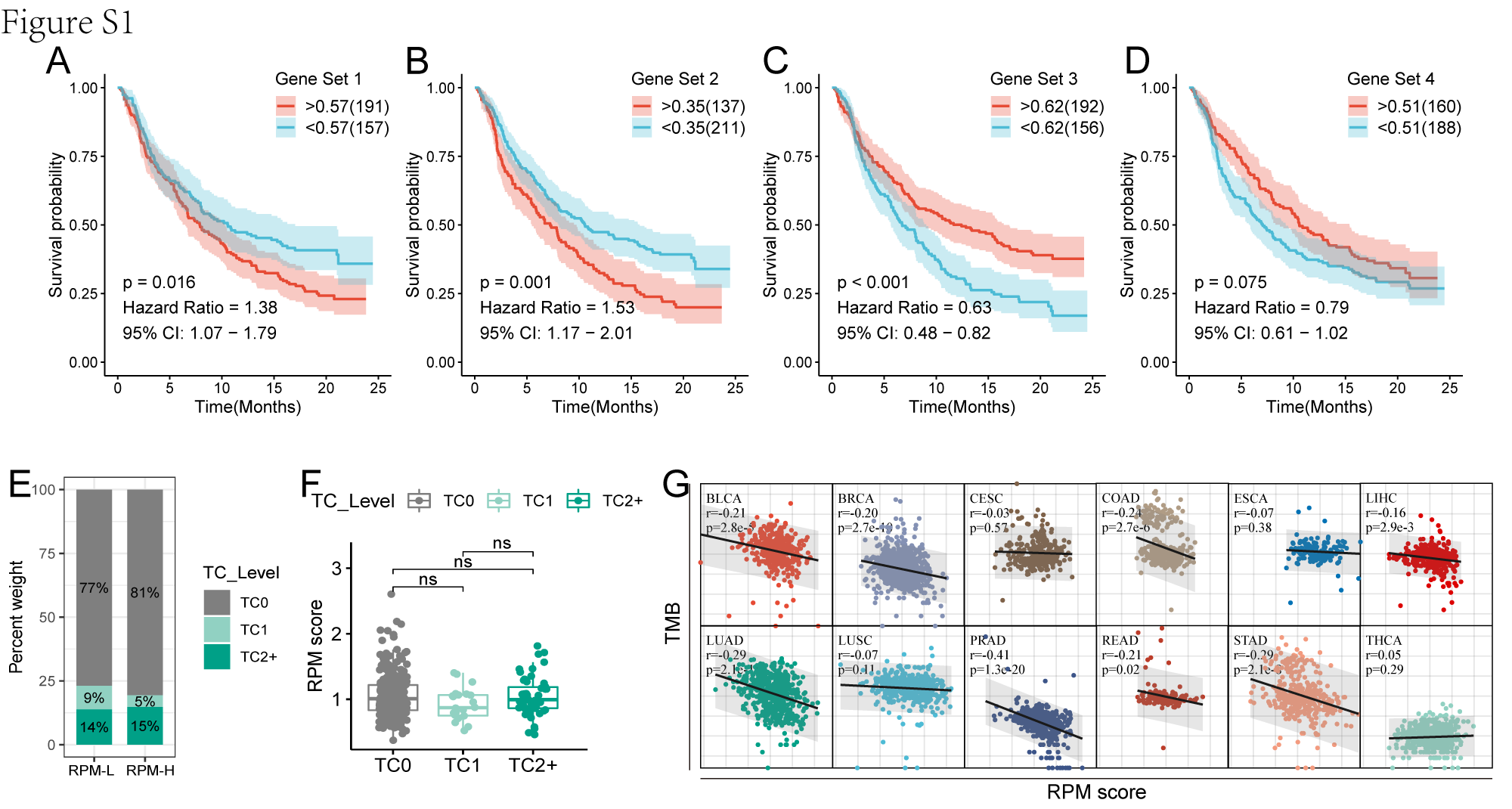

Supplement: Supplementary Figure 1 — Prognostic and immunologic relevance of RPM. (A–D) Kaplan–Meier analyses of Gene Sets 1–4 used to construct the RPM. (E) The TC levels were not significantly different between RPM-L and RPM-H groups. (F) RPM scores were not significantly different among different TC levels. (G) Correlation of RPM scores with TMB across 12 cancer types. ns, nonsignificant. [file Image_1.tif]
